# Informed consent in psychotherapy: a survey on attitudes among psychotherapists in Switzerland

**DOI:** 10.1186/s12910-021-00718-z

**Published:** 2021-11-12

**Authors:** Klara Eberle, Martin grosse Holtforth, Marc Inderbinen, Jens Gaab, Yvonne Nestoriuc, Manuel Trachsel

**Affiliations:** 1 Specialist Department for the Prevention of Eating Disorders, University Hospital Bern, Bern, Switzerland; 2 Department of Psychology, University of Bern, Bern, Switzerland; 3 Division of Psychosomatic Medicine, University Hospital Bern, Bern, Switzerland; 4 Clinical Psychology and Psychotherapy, Faculty of Psychology, University of Basel, Basel, Switzerland; 5 Helmut-Schmidt-University/University of the Federal Armed Forces Hamburg, Hamburg, Germany; 6 Systems Neuroscience, University Medical Centre Hamburg-Eppendorf, Hamburg, Germany; 7 Institute of Biomedical Ethics and History of Medicine, University of Zurich (UZH), Zürich, Switzerland; 8 Clinical Ethics Unit, University Hospital Basel (USB) and University Psychiatric Clinics (UPK), Basel, Switzerland

**Keywords:** Informed consent, Psychotherapy, Autonomy, Paternalism, Ethics, Expectations

## Abstract

**Background:**

The legal and ethical guidelines of psychological professional associations stipulate that informed consent by patients is an essential prerequisite for psychotherapy. Despite this awareness of the importance of informed consent, there is little empirical evidence on what psychotherapists’ attitudes towards informed consent are and how informed consent is implemented in psychotherapeutic practice.

**Methods:**

155 psychotherapists in Switzerland completed an online survey assessing their attitudes regarding informed consent.

**Results:**

Among the surveyed psychotherapists, there was a high consensus on important information that should be communicated to patients in the context of informed consent. Almost all psychotherapists rated confidentiality and its exemptions (95%) and self-determined decision-making (97%) as important. The importance to disclose information regarding fees and the empirical effectiveness of the provided treatment, were both seen as important by more than 80% of participants. The disclosure of personal information about the therapist was rated as important by 60%. Other aspects, which are not direct components of informed consent but rather overarching goals, were also evaluated rather homogeneously: self-determined decision making of the patient was rated as important by almost all of the surveyed psychotherapists (97%). The following components were also judged as important by a majority of the participants: promotion of hope (80%) and discussion of treatment goals (93%). Most psychotherapists described the implementation of informed consent as an ongoing process, rather than a one-time event during the first session of therapy. Therapists’ age, postgraduate training, treated patient group, and setting influenced attitudes towards informed consent.

**Conclusions:**

The present study shows that informed consent is perceived by psychotherapists as both a challenge and a resource. The implementation of informed consent in psychotherapy requires further research from a clinical and ethical perspective.

**Supplementary Information:**

The online version contains supplementary material available at 10.1186/s12910-021-00718-z.

## Background

Informed consent (IC) is a legal and ethical obligation and as such considered an important component of psychotherapy [1–5]. However, there is little empirical data on how IC is implemented and perceived in psychotherapeutic practice. Evidence so far shows that the meaning and implementation of IC are interpreted differently by individual psychotherapists [1, 6]. In addition, the format and processes pertaining to IC raised considerable questions and uncertainties in an explorative analysis of psychotherapists in training as some trainees reported not conducting IC or expressed mixed views, and uncertainties about who is responsible for IC [7].

The ethical and behavioral guidelines of various psychological professional associations show similar attitudes towards IC, in which patient autonomy is of highest priority [e.g., 8, 9]. Psychotherapists are to provide transparent information about treatment without being asked. Consequently, any paternalistic argumentation that certain information should be withheld from patients is rejected. For example, the APA calls upon its members to "[i]nform clients/patients as early as is feasible in the therapeutic relationship about the nature and anticipated course of therapy, fees, involvement of third parties, and limits of confidentiality and provide sufficient opportunity for the client/patient to ask questions and receive answers" (10.01) [8]. In previous studies, psychotherapists had been asked whether and how often they would discuss this specific information with their patients in the context of IC. The duty of confidentiality had been addressed by most therapists, but the topics of treatment alternatives and risks were only mentioned by a small proportion of the therapists [6, 10]. In the study by Somberg and colleagues [10], when asked why certain information had not been addressed by therapists, various explanations were given: (1) that the content in question was not relevant and not necessary for IC; (2) that they had too little information to adequately address a particular topic; (3) that they were not able to describe the procedure they were using; (4) that the patient was already well informed; or (5) that addressing certain topics would have a negative impact on the patient and/or the therapeutic relationship.

According to the APA [8], the IC should be carried out "as early as is feasible". This formulation already suggests that it often might not be possible in the context of psychotherapy to address all the necessary information for an IC during the first meeting [11]. Therefore, it has been argued that IC in psychotherapy should be an ongoing process rather than a one-time event at the beginning of therapy. In this way, IC would become a continuous exchange of information which accompanies the entire course of therapy [12–14]. Poppe named this a “procedural approach to informed consent […]. In this view, consequences and constituents of psychotherapy are only disclosed after some psychotherapy has transpired […]” [14]. However, this procedural approach to IC has been criticized as not being convincing:

Vaguely gesturing towards the “intrinsic uncertainty” of for example psychodynamic psychotherapy cannot serve as a justification for keeping patients out of the loop. It is correct that often informed consent needs to be a process rather than a one-stop shop […]. However, patients who invest their time and, many times, their money and who carry the risk of any damage that may result from treatments have the right to know several facts *before* committing to an intervention, and not some time along the way [15].

Psychotherapists often have different theoretical backgrounds. This manifests itself in different therapeutic directions and treatment rationales. This diversity makes a uniform implementation of IC difficult since the effects of psychotherapy can be understood differently depending on the treatment rationale, and information is therefore weighted differently. As a result, the information provided in the context of IC is dependent on the individual therapist [5]. Somberg and colleagues found significant differences in the weighting of individual components of IC depending on the therapeutic orientation [10]. Cognitive behavioral therapists rated information about the expected duration of treatment and possible treatment alternatives as more important and they indicated to address them more frequently in comparison to psychoanalytic and eclectic therapists. Croarkin and colleagues found that interpersonal therapists evaluated IC significantly more positively overall than psychoanalytic therapists [16]. In contrast, Dsubanko-Obermayr and Baumann did not find any significant differences between cognitive behavioral and psychodynamic therapists in terms of the general amount of information provided [6]. However, they also found significant differences in the weighting of individual topics. Cognitive behavioral therapists placed significantly more emphasis on communicating the methods used and the treatment goals, while psychoanalytic therapists considered the disclosure of financial agreements to be significantly more important.

These findings so far give rise to the impression that the surveyed psychotherapists have different attitudes regarding IC being associated with inconsistent IC practices in psychotherapy. However, the fact that certain information is not sufficiently discussed with patients might endanger their self-determined treatment decisions.

Therefore, in the present study, the aim was to assess psychotherapists’ attitudes towards IC. The following research question guided the survey: Which attitudes do psychotherapists have towards IC in psychotherapy? This research question was split into the following more concrete sub questions: (a) Which information do psychotherapists consider to be (how) important for IC? (b) Should psychotherapists omit certain information in the context of IC to minimize risk of negative consequences for the course of therapy? (c) Does IC influence patients' understanding of the disorder? (d) Can the mode of action of a psychotherapy be explained in advance, or can it only be experienced individually by patients during treatment? (e) Is IC understood as an ongoing process accompanying therapy or as a one-time event? (f) Do psychotherapists consider the expectations of a patient at the beginning of treatment to be influenced by IC?

## Methods

### Design and procedure

In the present study, an online survey was distributed among board-certified psychotherapists and postgraduate psychotherapy trainees working in Switzerland. The questionnaire was completed in German or French. Therefore, the questionnaire was developed in German and then translated into French by the first author of the paper (see Additional file 1 for the original questionnaire in German). It was corrected and checked for consistency by two bilingual persons. The online survey was implemented using the software SosciSurvey. The answers were collected completely anonymous.

### Survey questionnaire

The survey questionnaire was developed by the first and last author based on the research questions (see above), tested by a purposive pilot sample of 10 psychotherapists, and revised based on the respective feedback. The questionnaire consists of 20 items on attitudes towards IC. For example: “How important do you think it is to address the patients’ right to terminate the therapy in the IC”. The questions were answered by using a five-point Likert scale from “not important at all” to “very important” with the additional option "no answer”. The responses were statistically analyzed with IBM SPSS Statistics ® version 26.

### Recruitment and participants

Psychotherapists were recruited via email distributed by professional associations (Swiss Federation of Psychologists FSP; Swiss Federation of Applied Psychology SBAP) and several institutes for postgraduate psychotherapy training in Switzerland. The survey period lasted from September to November 2019.

To investigate a potential influence of the work context, the psychotherapists were asked whether they work with outpatients, inpatients, or day-clinic patients. Depending on the setting, different challenges could arise in the implementation of IC and, as a result, different therapeutic views on what is important regarding IC. Further, the education of participants was assessed, i.e., having a medical (medical psychotherapist) or psychological (psychological psychotherapist) background, or being specialized in a specific topic (e.g., specialized psychologist).

### Statistical analyses

Data were first analyzed descriptively for the whole sample. In a second step, it was examined whether responses differed significantly between subgroups. For this purpose, the overall sample was divided according to the variables “gender”, “age”, “patient group”, “setting”, and “postgraduate training status”. In the following statistical analysis, the collected Likert scale data were interpreted as parametric. Mean value comparisons between different subgroups were performed using *t*-tests for independent samples. Before *t*-tests were computed, the samples were checked for variance homogeneity. In the case of non-homogeneous variances, *Welch* tests were performed; in the case of homogeneous variances, *t*-tests were conducted [17]. The effect sizes were calculated using the software by *psychometrica.de*. To investigate the representativeness of the present sample, a *Chi-square* adjustment test was carried out for the individual categorical variables. It was tested whether the present sample differed significantly in its demographic characteristics from the total of psychotherapists in Switzerland. A membership statistic of psychotherapists by the Swiss Federation of Psychologists from 2018 and a structural survey of the Swiss Office for Labour and Social Policy Studies (BASS) on psychological psychotherapy in Switzerland from 2012 served as a basis for the calculation.

## Results

### Descriptives

A total of 155 subjects completed the questionnaire. In terms of gender and setting, the sample was comparable to the population of psychotherapists in Switzerland (gender χ^2^(1) = 1,331, *p* = 0.249; setting χ^2^(1) = 2,141, *p* = 0.343). However, there were significant differences in the variables of *patient group* and *education*. Therapists working with children and adolescents were overrepresented in the present sample (χ^2^(1) = 19,246, *p* < 0.000). No Chi-square adjustment test was carried out for the variables *postgraduate training* and *age*, as no up-to-date data was available for those variables. See detailed sociodemographic information in Table 1.

**Table 1:** Sociodemographics

Characteristics		N	%	M	SD	R
*Gender*						
Female		128	82.6			
Male		27	17.4			
*Age*				38.75	11.288 25–78	
20–40 years		104	67.1			
41–80 years		51	32.9			
*Education*						
Psychological psychotherapist		136	87.7			
Medical psychotherapist		6	3.9			
Others		12	7.7			
Not answered		1	0.6			
*Setting*						
Outpatient		110	71			
Partially inpatient		7	4.5			
Inpatient		37	23.9			
Not answered		1	0.6			
*Group of patients*						
Children and adolsecents		39	25.5			
Adults < 65 years		111	71.6			
Adults > 65 years		5	3.2			
*Postgraduate training*						
Completed		70	45.2			
In postgraduate training		85	54.8			

N total = 155

SD, standard deviation; R, range

### Attitudes towards IC

Therapists varied in their attitudes towards different aspects of IC (see Table 2). Almost all psychotherapists rated “confidentiality and its exemptions” (95%) and “self-determined decision-making” (97%) as important (“rather important” or “very important”). The importance to disclose information regarding “fees” and the “empirical effectiveness of the provided treatment,” were seen as important by about 80–85%. The disclosure of “personal information about the therapist” was rated as important by 60%.

**Table 2:** Descriptive statistics for the items: "How important do you consider addressing the following aspects in the informed consent?”

Item	M	SD	(1) (%)	(2) (%)	(3) (%)	(4) (%)	(5) (%)
Self-determined decision making	4.77	0.477	0	0	2.58	17.42	80
Confidentiality and its exemptions	4.76	0.523	0	0	4.52	14.84	80.65
Discussion of treatment goals	4.50	0.687	0	1.94	5.16	34.19	58.71
Promotion of hope	4.48	0.733	0	1.94	8.39	29.03	60.65
Right to therapy termination	4.41	0.804	0	3.23	10.32	28.39	58.06
Promotion of positive expectations	4.14	0.801	0.65	1.29	18.06	43.23	36.77
Frequency of consultations	4.08	0.837	0	5.16	15.48	45.16	34.19
Risiks	3.77	0.818	0	4.52	33.55	41.94	20
Fee	3.73	1.250	5.81	14.19	17.42	26.45	36.13
Treatment duration	3.55	0.839	0.65	9.68	34.19	44.52	10.97
Empirical effectiveness	3.36	0.904	1.94	15.48	34.84	40	7.74
Personal information about therapist	2.86	0.990	5.81	34.19	33.55	21.29	5.16

M, mean; SD, standard deviation

(1) not important at all; (2) not important; (3) neutral; (4) rather important; (5) very important

Other aspects, which are not direct components of IC but rather overarching goals, were evaluated rather homogeneously: “self-determined decision making of the patient” was rated as important by most of the surveyed psychotherapists (97%). Also, the following components were judged as important by most participants: “promotion of hope” (80%) and “discussion of treatment goals” (93%).

The variables *setting*, *patient group*, *age,* and *postgraduate training* status had a significant influence on the weighting of individual components. Inpatient psychotherapists assessed the right to discontinue therapy as significantly more important than outpatient therapists (d =  − 0.39; 95% CI [− 0.77, − 0.02]) as well as discussing treatment goals (d =  − 0.42; 95% CI [− 0.80, − 0.05]). Those who work with children and adolescents assessed the invoking of confidentiality and its limitations as significantly more important than therapists in the adult sector (d =  − 0.52; 95% CI [− 0.89, − 0.15]). There were also differences regarding the status of postgraduate training, where board-certified psychotherapists considered the discussion of fees (d =  − 0.58; 95% CI [− 0.90, − 0.26]) and personal information on the therapist (d =  − 0.34; 95% CI [− 0.66, − 0.02]) to be significantly more important while therapists in postgraduate training considered the promotion of positive expectations to be significantly more important (d = 0.33; 95% AI [0.01, 0.65). Similar age differences were found with the older therapists giving a significantly higher weight to fees (d = 0.50; 95% AI [0.16, 0.84]) and the provision of personal information on the therapist (d = 0.60; 95% AI [0.25, 0.94]). Detailed information is shown in Table 3.

**Table 3:** Mean value comparisons

Item	Gender Female versus male t (df)	Setting Outpatient versus inpatient t (df)	Patient group Children and adolescents versus adultst (df)	Postgraduate training Board-certified versus in postgraduate training t (df)	Age category 20–40 years versus 41–80 years t (df)
Confidentiality	− 0.18 (153)	− 0.43 (145)	3.99 (148)***	1.5 (153)	− 0.06 (153)
Right to therapy termination	1.36 (153)	2.58 (102)**	1.7 (89)	− 0.39 (153)	− 1.06 (153)
Empirical effectiveness	− 0.53 (153)	− 0.65 (145)	− 0.37 (148)	− 0.59 (153)	− 0.3 (153)
Risks	− 0.29 (153)	1.59 (88)	− 1.02 (148)	− 0.23 (127)	− 0.87 (81)
Personal information	− 0.4 (153)	− 1.83 (145)	− 0.38 (148)	2.14 (153)**	− 3.24 (86)***
Frequency of meetings	1.55 (33)	0.69 (145)	0.37 (148)	− 0.75 (153)	0.88 (153)
Treatment duration	− 0.51 (153)	1.34 (145)	0.65 (148)	− 0.17 (153)	− 0.35 (153)
Fee	− 0.06 (153)	− 1.97 (48)**	1.24 (84)	3.69 (153)***	− 3.05 (112)***
Promotion of hope	0.89 (153)	0.97 (145)	− 0.44 (148)	− 1.98 (153)	1.57 (153)
Promotion of positive expectations	0.75 (153)	1.18 (145)	− 1.45 (148)	− 2.03 (153)**	1.77 (153)
Discussion of treatment goals	1.06 (153)	2.53 (78)**	− 0.8 (148)	0.06 (153)	− 0.17 (153)
Self-determined decision making	0.85 (153)	0.52 (145)	− 0.67 (148)	− 0.07 (153)	− 1.77 (123)
Promotion of positive expectation through IC	0.38 (145)	0.9 (138)	0.44 (141)	− 0.41(145)	− 0.54 (145)
To not address risks	− 1.35 (31)	− 1.63 (144)	− 1.89 (90)	1.24 (152)	− 1.17 (83)
To not address alternatives	− 0.51 (147)	− 0.44 (80)	− 0.07 (143)	1.24 (126)	− 1.2 (147)
IC and understanding of the disorder	0.18 (136)	1.27 (129)	− 0.79 (131)	− 1.04 (106)	0.71 (65)
Liberty to implement IC as I see fit	− 0.07 (150)	− 3.5 (143)***	− 1.48 (145)	2.41 (150)**	− 2.37 (150)**
In my practice, I have enough time resources to implement IC as I see fit	− 1.62 (151)	− 4.49 (51)***	0.67 (146)	3.94 (151)***	− 4.29 (134)***
The mode of action of a therapy cannot be explained in advance. It can only be experienced individdually by patients during treatment	− 0.7 (151)	− 0.54 (143)	− 0.84 (147)	− 0.07 (151)	− 0.95 (151)
IC is an ongoing process during the whole course of therapy. Therefore, IC is never completely terminated	0.67 (148)	− 0.37 (140)	0.54 (143)	− 0.79 (148)	− 0.27 (75)

df, degrees of freedom; IC, informed consent

**p < 0.05; ***p < 0.01

92% of the participants understood IC rather as an ongoing process than a one-time event; no therapist rejected this completely (see Table 4). There were no significant differences between the different subgroups in this regard. Also, the assumption that the patient's expectations could be influenced by the IC was agreed to by most participants with 79% answering “rather agree” or “fully agree” and only 4% rejecting it rather or completely (see Table 4). The responses did not differ significantly between the individual subgroups. Addressing potential risks and treatment alternatives were both agreed to by 74% of the respondents (see Table 4). This assessment did not differ significantly according to gender, setting, patient group, age, or postgraduate training status. The statement whether IC could help in the understanding of the disorder did not give a clear picture: 35% of the respondents chose the answer option "neutral" or "no answer" (see Table 4). There was no significant difference between the subgroups. 86% of the respondents rather or fully agreed to having enough liberty in their daily practice to implement IC as they see fit (see Table 4). However, there were significant differences depending on the setting, postgraduate training status, and age. Outpatient therapists assessed their liberty to implement IC as they see fit as significantly greater than in-patient practitioners (d = 0.66; 95% CI [0.28, 1.04]). The same applied to therapists who had completed postgraduate training compared with those who were currently still in postgraduate training (d = 0.38; 95% CI [0.06], 0.71) and to the older age category compared to the younger (d = 0.41; 95% CI [0.07, 0.75]). When it comes to sufficient time resources to implement IC as they see fit, most therapists indicated to have enough time (76%) (see Table 4). 14%, on the other hand, did less or not at all agree thus stating that they did not have sufficient time resources to implement IC as they see fit. There were also significant differences in this item depending on the setting, level of postgraduate training, and age. Outpatient psychotherapists stated that they had significantly more time resources at their disposal for implementing IC as they see fit than those working in inpatient settings (d = 0.98; 95% CI [0.59, 1.37]). The same was true for therapists who are board-certified compared to participants in postgraduate training (d =  − 0.63; 95% CI [− 0.71, − 0.06]) and for the older age group compared to the younger one (d = 0.66; 95% CI [0.32, 1.01]) (see Table 4).

**Table 4:** Descriptive statistics for the items: "How much do you agree with the following statements on informed consent based on your personal experience?”

Item	M	SD	(1)	(2)	(3)	(4)	(5)	(6)
IC is an ongoing process during the whole course of therapy. Therefore, IC is never completely terminated	4.41	0.636	0%	1.29%	3.87%	45.81%	45.81%	3.23%
In my practice, I have the liberty to implement IC as I see fit	4.32	0.925	1.29%	6.45%	3.87%	34.19%	52.26%	1.94%
In my practice, I have enough time resources to implement IC as I see fit	4.02	1.121	3.23%	10.97%	8.39%	34.19%	41.94%	1.29%
Patients’ outcome expectations are influenced by the IC	4.01	0.767	1.29%	2.58%	11.61%	57.42%	21.94%	5.16%
IC at the beginning of therapy influences how patients experience their psychological suffering	3.47	0.998	4.52%	9.68%	23.87%	41.29%	9.68%	10.97%
The mode of action of therapy cannot be explained in advance. It can only be experienced individdually by patients during treatment	2.93	1.145	5.81%	41.29%	14.19%	28.39%	9.03%	1.29%
I advise against addressing risks at the beginning of therapy	2.09	1.006	30.32%	43.87%	12.26%	11.61%	1.29%	0.65%
I advise against addressing treatment alternatives which I do not practice at the beginning of therapy	2.06	1.028	30.32%	44.52%	9.03%	9.68%	2.58%	3.87%

M, mean; SD, standard deviation; IC, informed consent

(1) do not agree at all; (2) rather not agree; (3) neutral; (4) rather agree; (5) fully agree; (6) no answer

55% of the participants didn’t agree at all or rather not agreed with the statement that the mode of action of psychotherapy must be individually experienced and cannot be explained in advance whereas 37% of the therapists rather or fully agreed. The responses did not differ significantly between the different subgroups (see Table 4).

## Discussion

### Focus on information: not everything seems equally important

Information about the duty of *confidentiality* and information about the *right to discontinue therapy* were considered the most important elements of IC (from amongst the elements given to the participants to rate). Seen as least important from amongst the elements given to the participants to rate were informing about *empirical effectiveness* of the treatment and *personal information about the therapist*. These findings coincided with results of previous studies. Somberg and colleagues also concluded that confidentiality was the most important element of IC [10]. Croarkin and colleagues [16] and Dsubanko-Obermayr and Baumann [6] also found personal information about the therapist and empirical effectiveness to be rated as less important. However, in the present study, the differences between the elements of IC and the differences between therapists regarding their respective attitudes were relatively small (see standard deviations in Tables 2, 3).

Nevertheless, significant differences were found between the various therapist subgroups. In contrast to previous studies, the present study found a significant influence of the setting, patient group, age, and postgraduate education status on the reported attitudes and experiences (see next subsection below). This raises the question of whether there is a greater awareness of the importance of IC among psychotherapists nowadays than at the time of the study by Dsubanko-Obermayr and Baumann [6]. One possible explanation for the greater consensus among therapists could be the development and binding character of ethical guidelines for the implementation of IC.

### Focus on therapists: not the same for everyone

Children- and youth-therapists considered the discussion of confidentiality and its exceptions with their clients as significantly more important than therapists of adults. One possible explanation for this difference could be that the influence of third parties (such as parents, teachers, etc.) is greater in underage patients and that therapists are more frequently obliged to inform them about the therapy and consequently must extend the confidentiality obligation to include the relevant third parties.

Addressing the *fee* and *personal information about the therapist* were of differential importance, with older therapists (41–80 years) and those who have completed postgraduate training considering both aspects to be significantly more important than younger therapists or those in postgraduate training. This could be because younger therapists and those in postgraduate training more often work in inpatient settings than older therapists or those who have completed continuing education. The different importance of personal information could also be related to the setting. In an inpatient setting, personal information about the therapist could be considered less important than in a long-term outpatient therapy relationship due to the shorter duration of treatment as well as the involvement of other professionals within larger multimodal treatments.

Furthermore, inpatient therapists considered the *right to discontinue therapy* and the *discussion treatment goals* to be significantly more important issues than their outpatient colleagues. One reason for this could be that inpatient treatment is more often carried out in the context of an extrinsic patient motivation and it is therefore important to stress the right to discontinue therapy – or to appeal in the case of an involuntary hospitalization. In addition, the usually shorter duration of treatment in an inpatient setting makes it seem more important to discuss treatment goals, since concrete criteria for leaving the clinic are often defined at the beginning of an inpatient treatment.

### Informed consent as an ongoing process or one-time event?

A large majority of the therapists surveyed (92%) understood IC as an ongoing process that accompanies the course of therapy and not as a one-time event at the beginning of treatment. This attitude is in line with the view of various researchers [13]. However, it is important to note that from a legal and ethical point of view, the procedural form of IC cannot replace formal consent at the beginning of therapy [15]. IC as an ongoing process should therefore be seen as complementary to formal consent at the beginning of therapy [11]. This raises the question of which components of IC should be addressed by default at the beginning of treatment and which can only be addressed during the process.

There is existing literature clearly arguing that patients “should receive information regarding the risks and benefits of treatment as well the alternatives that exist to the treatment being recommended. This discussion would include information regarding the diagnosis, the variable course of illness expected, the potential for worsening with or without treatment, duration of treatment as well as the existing treatment alternatives and their empirical evidence” [18, p. 262; see also 5, 19]. Different professional organizations argue for various additional aspects which should be included in a psychotherapeutic informed consent. The American Psychological Association recommends sharing further details such as “…fees, involvement of third parties, and limits of confidentiality” [8]. However, the question which additional components informed consent for psychotherapy should entail at the beginning of psychotherapy requires further investigation and discussion.

“By having a forthright discussion about treatment goals, expectations, pitfalls and treatment options available to them, the therapist grounds the treating relationship in honesty and adheres to their fiduciary role.” [18, p. 264]. In a survey on the negative effects of psychotherapy, Crawford and colleagues [20] “had two crucial findings that speak to the process of educating patients about their treatments […]. They observed that patients who could not describe what therapy they had been given were more likely to have experienced negative effects than those who could clearly state the type of therapy received (Odds Ratio (OR) = 1.51, 95% Confidence Interval (CI) 1.22–1.87). Additionally, those who endorsed that they had not received enough information about therapy from their provider prior to commencing treatment were also more likely to have had negative results than those who felt that they had received enough information (OR = 0.65, 95% CI 0.54–0.79).” [18, p. 264].

Beyond the initial session, further discussions regarding informed consent need to occur at other points in treatment depending on the patient’s progress or clinical material encountered. This may be particularly germane depending on the psychotherapy employed. For example, this necessity may be different between a manualized brief-psychotherapy (e.g., cognitive-behavioral therapy or interpersonal psychotherapy) with a more predictable course versus a long-term more open-ended treatment (e.g., insight-oriented psychodynamic therapy) that may be more likely to take an unanticipated direction […]. Patients may need to have both written and verbal sharing of information about their care to make this process more efficient and to ensure that they understand the benefits, risks and alternatives that may come up at certain points in care. Certainly, if the patient’s condition were to change and treatment course were altered the patient should be informed of additional treatment alternatives that may exist, including for example other psychotherapies or pharmacotherapies that may apply [18, p. 264; see also 2].

### Liberty and time resources of psychotherapists to implement IC as they see fit

More than 3 out of 4 participants agreed having sufficient liberty and time resources to implement the content of IC as they saw fit. Thus, most psychotherapists seem to be satisfied with the liberty and resources available to them to individually implement IC. However, there were differences between the sub-groups. Outpatient therapists indicated to have significantly more liberty than their inpatient colleagues. Possible reasons for these differences could be a different structure of the everyday work, whereby in the in-patient setting there is more time pressure, and the therapists consequently have less time to implement IC. In addition, there could be more guidelines for initial consultations in the inpatient setting. Another possible cause of the observed differences may be that crisis situations, in which patients' ability to make decisions may be limited, occur more frequently in the inpatient setting. Under these conditions, adequate education of patients may be more demanding and may require more time. This could result in a double challenge in the inpatient setting: patients are more often restricted in their current decision-making capacity and the time for IC is shorter. The implementation of IC in an inpatient setting should therefore be examined more closely from an ethical perspective in further studies.

Significant differences in the assessment of one's own liberty to implement IC and time resources were also found for the status of postgraduate training and age. Therapists who had completed postgraduate training and those between 41 and 80 years of age reported significantly more often that they had sufficient liberty and time resources to implement IC than younger therapists (20–40 years) and those in postgraduate training. This could be because older therapists with more experience have been increasingly developing their own strategies for dealing with IC and consequently feel more confident and experience less time pressure when implementing them.

### Limitations

Since, to our knowledge, no psychometrically validated questionnaires are available to investigate the present questions, a specific questionnaire has been developed and used in this study for the first time. Limitations of the questionnaire itself have been identified, which should be revised for future use. For example, the participants were asked the following question: “Is IC understood as an ongoing process accompanying therapy or as a one-time event”? This question leaves the participants no option to state that informed consent must be both, (a) a mandatory information at the beginning of psychotherapy on diagnosis, kind of treatment and alternative treatment options, risks and side-effects, costs, or format/duration; and (b) further ongoing information and discussion during the therapy process, e.g., when actual side-effects become obvious or can be anticipated before certain interventions are initiated. Furthermore, a future study could add questions on psychotherapist´s experiences and on suggestions about how to take advantage of informed consent to maximize positive expectations, minimize nocebo-effects, support psychoeducation, or with regard to supporting normalization and validation.

Due to the recruitment procedure of psychotherapists via email distributed by two professional associations (Swiss Federation of Psychologists FSP; Swiss Federation of Applied Psychology SBAP) and nine institutes for postgraduate psychotherapy training in Switzerland, it cannot be inferred how many potential participants have received and read the invitation email. This neither allows for calculating a response rate. The present sample is also not representative for all psychotherapists in Switzerland since child and youth therapists and psychotherapists in postgraduate training were over-represented in the present sample, which could have led to biased findings. Due to the ethical significance of IC, a certain influence of social desirability in the responses of therapists cannot be ruled out.

## Conclusion

In the present survey, psychotherapists in Switzerland rated information about autonomous decision making and treatment confidentiality as important in IC for psychotherapy. In accordance with data from their colleagues in the United Kingdom [7] and Austria [6], they considered personal information about the therapist and information about treatment effectiveness to be less important. Furthermore, they seem to consider IC for psychotherapy as resource to influence patients’ treatment expectations and illness/symptom perception. This is an important finding. However, the findings do not allow any concrete interpretation with regard to how exactly psychotherapists could benefit from the informed consent process to influence patients’ treatment expectations, illness/symptom perception, to minimize nocebo-effects, support psychoeducation, or with regard to normalization and validation. Because there might be an underused potential in order to increase effectiveness of psychotherapy, these aspects should be assessed in future studies.

However, aspects like explaining the mode of action of psychotherapy or having enough time and resources to perform IC were seen as more of a challenge. Especially, the question about how the mode of action of psychotherapy should be communicated to patients before or/and during therapy must be further studied. A differentiated picture of IC resulted according to treatment settings and psychotherapists’ training status. Future studies might investigate these differing aspects of IC, their perceptions within the professional community, as well as their potential to influence important aspects of the treatment itself, such as expectations management, efficacy, and risk perception.

## Supplementary Information

**Additional file 1**. Survey questionnaire in German.

## Data Availability

The dataset is available from the corresponding author on reasonable request. For the survey questionnaire, see Additional file 1.

## Abbreviation

IC: Informed consent
