# Supplementary material for: Informed consent in psychotherapy: a survey on attitudes among psychotherapists in Switzerland
Source: BMC Med Ethics. 2021 Nov 12;22:150. doi: 10.1186/s12910-021-00718-z (PMC8588676; doi:10.1186/s12910-021-00718-z)
Supplement: Supplementary file 1 — Additional file 1. Survey questionnaire in German. [file 12910_2021_718_MOESM1_ESM.pdf]

Fragebogen

Fragebogen deutsch

0% ausgefüllt

Sehr geehrte Teilnehmerin, sehr geehrter Teilnehmer

Vielen Dank, dass Sie sich die Zeit nehmen, einige Fragen zu beantworten.
Wir stellen Ihnen diese Fragen im Rahmen einer Masterarbeit in klinischer Psychologie an der Universität Bern.

Dabei interessieren wir uns für Ihre persönlichen Erfahrungen mit dem **Informed Consent** (dt. **Informierte Einwilligung**) in Ihrer therapeutischen Arbeit mit Klient*innen.
Eine kurze Beschreibung des Informed Consent folgt auf der nächsten Seite.

Aufgrund der Erfahrungen und Ratschläge von medizinischen und psychologischen Fachpersonen möchten wir ein **Fazit für die Praxis** entwickeln. Dieses Fazit soll Psychotherapeut*innen in Weiterbildung bei der Umsetzung des Informed Consent unterstützen, indem sie von den Erfahrungen und Tipps anderer Therapeut*innen lernen können.

9% ausgefüllt

Datenschutzbedingungen

Das Ausfüllen des Fragebogens wird ca. 10 Min dauern. Ihre Antworten werden vollständig anonymisiert auf dem Server von SoSci Survey (Werkzeug für Onlinebefragungen) gespeichert und können zu keinem Zeitpunkt persönlich zugeordnet werden.

Nur das Studienteam hat Zugriff auf diese Daten, die sicher aufbewahrt und gemäss Richtlinien des Schweizerischen Nationalfonds (SNF) nach zehn Jahren gelöscht werden.

Sie haben jederzeit die Möglichkeit das Ausfüllen des Fragebogens abzubrechen, ohne dass Ihnen deswegen Nachteile entstehen. In diesem Fall werden die bis dahin erhobenen Daten direkt gelöscht. Bei Fragen oder Unsicherheiten bzgl. Datenschutz dürfen Sie mich gerne kontaktieren.

Herzlichen Dank für Ihre Mithilfe.

Klara Eberle

Indem Sie auf **Weiter** klicken, erklären Sie sich mit den Datenschutzbedingungen einverstanden.

18% ausgefüllt

Was ist mit Informed Consent gemeint?

Ein Patient benötigt zu Beginn einer Behandlung bestimmte Informationen. Nur wenn er über ausreichende Informationen verfügt, ist es ihm möglich, **informiert** in eine Behandlung **einzuwilligen**.

In diesem Fragebogen interessieren wir uns für **Ihre Meinungen und Erfahrungen** mit dem Informed Consent in Ihrer therapeutischen Tätigkeit. Ihre Antworten können dementsprechend nicht richtig oder falsch sein.

27% ausgefüllt

Zu Beginn bitten wir Sie um einige Angaben zu Ihrer Person:

Welches Geschlecht haben Sie?

- ◯ Weiblich
- ◯ Männlich
- ◯ Anderes

Wie alt sind Sie?

Ich bin ____ Jahre alt

Haben Sie eine abgeschlossene psychotherapeutische Weiterbildung?

- ◯ Ja
- ◯ Nein, ich bin noch in Weiterbildung

Welchen Berufsabschluss haben bzw. machen Sie?

- ◯ Fachpsycholog*in Psychotherapie
- ◯ Fachpsycholog*in mit anderem Schwerpunkt als Psychotherapie
- ◯ Facharzt/ärztin Psychotherapie
- ◯ Anderer Berufsabschluss: ____

In welchem Setting arbeiten Sie aktuell?

Wenn Sie in verschiedenen Settings tätig sind, wählen Sie bitte das häufigste aus.

- ◯ Stationär
- ◯ Teilstationär
- ◯ Ambulant

Mit welcher Patientenaltersgruppe arbeiten Sie?

Wenn mehrere der Patientengruppen für Sie zutreffen, wählen Sie bitte die häufigste Gruppe aus.

- ◯ Kinder und Jugendliche
- ◯ Erwachsene zwischen 18 und 65 Jahren
- ◯ Erwachsene über 65 Jahre

36% ausgefüllt

Als Einstieg einige Fragen zu Ihrer persönlichen Sichtweise auf den Informed Consent:

*Für wie wichtig halten Sie das Ansprechen folgender Themen für einen Informed Consent Ihrer Klient*innen:*

Die Schweigepflicht des Therapeuten und ihre Ausnahmen

- Sehr wichtig
- Eher wichtig
- Neutral
- Wenig wichtig
- Überhaupt nicht wichtig

Das Recht des Patienten auf Beendigung der Therapie

- Sehr wichtig
- Eher wichtig
- Neutral
- Wenig wichtig
- Überhaupt nicht wichtig

Die empirische Wirksamkeit der von Ihnen angewandten Behandlungsmethoden

- Sehr wichtig
- Eher wichtig
- Neutral
- Wenig wichtig
- Überhaupt nicht wichtig

Mögliche Risiken einer Behandlung

- Sehr wichtig
- Eher wichtig
- Neutral
- Wenig wichtig
- Überhaupt nicht wichtig

Persönliche Informationen über Sie als Therapeut*In

Sehr wichtig
Eher wichtig
Neutral
Wenig wichtig
Überhaupt nicht wichtig

Die Sitzungsfrequenz

Sehr wichtig
Eher wichtig
Neutral
Wenig wichtig
Überhaupt nicht wichtig

Die vorgesehene Dauer der Behandlung

Sehr wichtig
Eher wichtig
Neutral
Wenig wichtig
Überhaupt nicht wichtig

Informationen über das Honorar

Sehr wichtig
Eher wichtig
Neutral
Wenig wichtig
Überhaupt nicht wichtig

45% ausgefüllt

*Für <u>wie wichtig</u> erachten Sie des Weiteren für einen Informed Consent ihrer Klient*innen:*

Die Förderung von Hoffnung und Zuversicht für die Zukunft

- Sehr wichtig
- Eher wichtig
- Neutral
- Wenig wichtig
- Überhaupt nicht wichtig

Die Förderung positiver Erwartungen bezüglich der bevorstehenden Behandlung

- Sehr wichtig
- Eher wichtig
- Neutral
- Wenig wichtig
- Überhaupt nicht wichtig

Die Vereinbarung konkreter Behandlungsziele

- Sehr wichtig
- Eher wichtig
- Neutral
- Wenig wichtig
- Überhaupt nicht wichtig

Das selbstbestimmte Entscheiden des Patienten

- Sehr wichtig
- Eher wichtig
- Neutral
- Wenig wichtig
- Überhaupt nicht wichtig

55% ausgefüllt

Nun interessieren uns auch Ihre persönlichen Erfahrungen mit dem Informed Consent und mit Patientenerwartungen zu Beginn einer Behandlung.

Wie sehr stimmen Sie aufgrund Ihrer persönlichen Erfahrungen den folgenden Aussagen zu bzw. lehnen diese ab?

Die Erwartungen eines Patienten an den Erfolg der Behandlung werden durch den Informed Consent beeinflusst.

- Stimme voll zu
- Stimme eher zu
- Neutral
- Stimme eher nicht zu
- Stimme gar nicht zu
- *Kann ich nicht beurteilen*

Ich rate davon ab, zu Beginn einer Therapie die möglichen Risiken (wie z.B. eine temporäre Verschlechterung des Zustandes) anzusprechen. Dies verschlechtert die Hoffnung des Patienten auf Besserung.

- Stimme voll zu
- Stimme eher zu
- Neutral
- Stimme eher nicht zu
- Stimme gar nicht zu
- *Kann ich nicht beurteilen*

Ich rate davon ab, zu Beginn über andere Behandlungsmöglichkeiten, die ich selbst nicht praktiziere, zu berichten. Dies senkt das Vertrauen des Patienten in mich als Therapeut*in.

- Stimme voll zu
- Stimme eher zu
- Neutral
- Stimme eher nicht zu
- Stimme gar nicht zu
- *Kann ich nicht beurteilen*

Die Art und Weise, wie ein Patient sein psychisches Leiden wahrnimmt, wird durch den Informed Consent zu Beginn der Behandlung beeinflusst.

- Stimme voll zu
- Stimme eher zu
- Neutral
- Stimme eher nicht zu
- Stimme gar nicht zu

Kann ich nicht beurteilen

64% ausgefüllt

Wie erleben Sie die praktische Umsetzung des Informed Consent in Ihrer therapeutischen Tätigkeit?

Wie sehr stimmen Sie den folgenden Aussagen zu bzw. lehnen diese ab?

In meinem Praxisalltag habe ich den Spielraum, den Informed Consent inhaltlich so umzusetzen, wie ich das wünsche.

- Stimme voll zu
- Stimme eher zu
- Neutral
- Stimme eher nicht zu
- Stimme gar nicht zu

Kann ich nicht beurteilen

In meinem Praxisalltag habe ich die zeitlichen Ressourcen, den Informed Consent so umzusetzen, wie ich das wünsche.

- Stimme voll zu
- Stimme eher zu
- Neutral
- Stimme eher nicht zu
- Stimme gar nicht zu

Kann ich nicht beurteilen

INFORMED CONSENT IN DER PSYCHOTHERAPIE

73% ausgefüllt

Wie sehr stimmen Sie, aufgrund Ihrer persönlichen Erfahrungen, den folgenden Aussagen zu, bzw. lehnen Sie diese ab?

Die Art und Weise wie eine Therapie wirkt, kann nicht im Voraus erklärt werden. Sie kann nur vom Patienten selbst erlebt werden im Verlauf seiner Behandlung.

- stimme voll zu
- Stimme eher zu
- Neutral
- Stimme eher nicht zu
- Stimme gar nicht zu
- *Kann ich nicht beurteilen*

Der Begriff des Informed Consent wird bzw. wurde mir im Rahmen meiner therapeutischen Weiterbildung nähergebracht.

- stimme voll zu
- Stimme eher zu
- Neutral
- Stimme eher nicht zu
- Stimme gar nicht zu
- *Kann ich nicht beurteilen*

Die Umsetzung des Informed Consent ist bzw. war Teil meiner therapeutischen Weiterbildung.

- stimme voll zu
- Stimme eher zu
- Neutral
- Stimme eher nicht zu
- Stimme gar nicht zu
- *Kann ich nicht beurteilen*

Einen Informed Consent zu erlangen, bedeutet ein anhaltendes Abgleichen mit dem Patienten, welches über den gesamten Therapieverlauf andauert. Deshalb ist der Informed Consent nie ganz abgeschlossen.

- stimme voll zu
- Stimme eher zu
- Neutral
- Stimme eher nicht zu
- Stimme gar nicht zu
- *Kann ich nicht beurteilen*

82% ausgefüllt

Die Umsetzung des Informed Consent in einer Psychotherapie kann eine herausfordernde Angelegenheit darstellen.
Wann erleben Sie persönlich in Ihrer therapeutischen Arbeit den Informed Consent als Herausforderung?

Bitte notieren Sie Ihre Gedanken dazu hier

Was haben Sie persönlich in solchen Situationen jeweils als hilfreich empfunden, was Sie anderen Therapeut*innen in Weiterbildung raten könnten?

Bitte notieren Sie Ihre Gedanken dazu hier

Wir möchten uns herzlich für Ihre Mithilfe bedanken.

Falls Sie Fragen oder Interesse an den Befunden unserer Studie haben, könnten Sie mich gerne kontaktieren unter klara.eberle@students.unibe.ch

Ihre Antworten wurden gespeichert, Sie können das Browser-Fenster nun schließen.

B.Sc. Klara Eberle, Universität Bern – 2019
